# Influence of *DAT1* Promotor Methylation on Sports Performance

**DOI:** 10.3390/genes12091425

**Published:** 2021-09-16

**Authors:** Anna Grzywacz, Krzysztof Chmielowiec, Agnieszka Boroń, Monika Michałowska-Sawczyn, Jolanta Chmielowiec, Grzegorz Trybek, Bożena Mroczek, Katarzyna Leźnicka, Paweł Cieszczyk, Jolanta Masiak

**Affiliations:** 1 Independent Laboratory of Health Promotion of the Pomeranian Medical University in Szczecin, 11 Chlapowskiego St., 70-204 Szczecin, Poland; 2 Department of Hygiene and Epidemiology, Collegium Medicum, University of Zielona Góra, 28 Zyty St., 65-046 Zielona Góra, Poland; chmiele@vp.pl (K.C.); chmiele1@o2.pl (J.C.); 3 Department of Clinical and Molecular Biochemistry, Pomeranian Medical University, 72 Powstańców Wielkopolskich St., 70-111 Szczecin, Poland; binia@pum.edu.pl; 4 Faculty of Physical Education, Gdansk University of Physical Education and Sport, 1 K.Górskiego St., 80-336 Gdansk, Poland; monikamichalowska@op.pl (M.M.-S.); katarzyna.leznicka@awf.gda.pl (K.L.); cieszczyk@poczta.onet.pl (P.C.); 5 Department of Oral Surgery, Pomeranian Medical University in Szczecin, 72 Powstańców Wlkp. St., 70-111 Szczecin, Poland; g.trybek@gmail.com; 6 Department of Human Sciences in Medicine, Pomeranian Medical University in Szczecin, 11 Chlapowskiego St., 70-204 Szczecin, Poland; b_mroczek@data.pl; 7 II Department of Psychiatry and Psychiatric Rehabilitation, Medical University of Lublin, 1 Głuska St., 20-059 Lublin, Poland; jolantamasiak@wp.pl

**Keywords:** BDNF, genes, athletes, personality, rs10767664, rs2030323

## Abstract

In the mammalian genome, DNA methylation is an epigenetic mechanism involving the transfer of a methyl group onto the C5 position of the cytosine to form 5-methylcytosine. DNA methylation regulates gene expression by recruiting proteins involved in gene repression or by inhibiting the binding of transcription factors (TFs) to DNA. As there are still many questions concerning the role of methylation in creating personality, we concentrated on searching for such associations. The research group was 100 sports male subjects (mean age = 22.88, SD = 6.35), whereas the control group included 239 healthy male volunteers matched for age (mean age = 21.69, SD = 3.39), both of European origin. The methods used in our research were as follows: DNA isolation, methylation-specific PCR, sequencing chromatophores, all conducted according to the manufacturer’s procedure. To evaluate personality traits, the NEO Five-Factor Personality Inventory (NEO-FFI) and STAI Inventory were used. We observed the existence of a statistically significant correlation for all the aspects of personality covered and CpG islands’ methylation. Nonetheless, we think that the tested group and the number of tested promotor islands in the *DAT1* gene are still too small to make explicit conclusions, so it needs further profound analysis.

## 1. Introduction

### 1.1. DNA Methylation

In the mammalian genome, DNA methylation is an epigenetic mechanism involving the transfer of a methyl group onto the C5 position of the cytosine to form 5-methylcytosine. DNA methylation regulates gene expression by recruiting proteins involved in gene repression or by inhibiting the binding of transcription factors (TFs) to DNA. One distinct feature of transcription factors is that they have DNA-binding domains that give them the ability to bind to specific sequences of DNA called enhancer or promoter sequences. Some transcription factors bind to a DNA promoter sequence near the transcription start site and help form the transcription initiation complex. Other transcription factors bind to regulatory sequences, such as enhancer sequences, and can either stimulate or repress transcription of the related gene. These regulatory sequences can be thousands of base pairs upstream or downstream from the gene being transcribed. Regulation of transcription is the most common form of gene control [1]. DNA methylation in the mammalian genome predominantly occurs on cytosine in the context of the 5′-CpG-3′ dinucleotides; this is the only type of epigenetic modification to change the DNA molecule directly. Stretches of GC-rich sequences in the genome called CpG islands (CGIs) that are associated with open transcriptionally competent chromatin structures were discovered in gene promoters [2]. Promoters play an essential role in understanding the transcriptional mechanisms of genes. CpG islands located within promoter regions appear to create a more conducive chromatin state that favors transcription or allows gene expression silencing through intensive CpG methylation [3]. The relevance of GC content and CpG dinucleotide concentration to the regulation of gene activity points to its physiological significance. The DNA methylation pattern established during the development and differentiation is preserved with high fidelity during cell division by DNA methyltransferases (DNMTs). DNMTs are highly expressed in developing tissues, but their activity declines during differentiation in all tissues except the brain, wherein they are expressed throughout the lifetime of the brain [4,5]. Dynamic DNMT activity in the brain is essential for synaptic plasticity and memory formation [6,7]. The environment is a potent genetic modifier, influencing gene expression via epigenetic mechanisms. Ubiquitous epigenetic mechanisms represent an essential element of normal development and maturation. Aberrant epigenetic processes can cause maladaptive changes (gene dysregulations and dysfunctions) and consequently lead to disease. In the brain, in contrast to somatic tissues, epigenetic processes remain active throughout the lifespan: they are ultimately involved in maintaining brain functions, enabling adaptive plasticity and the ability to accommodate varying environmental challenges [8]. The fidelity of epigenetic processes is critical for the human brain since its development creates enormously complex biological patterning. Consequently, it is most susceptible to aberrant activity of epigenetic modifiers: epigenetic dysregulation is implicated in the pathogenesis of a variety of brain-related diseases, including mental retardation and complex psychiatric disorders [9].

Physical exercises have a beneficial influence on both, brain and body, and particularly on skeletal muscles. They increase the effectiveness of muscles metabolism, improve the biological functions of mitochondria, adjust the transformation of muscle fiber types, and increase muscle strength. Currently, conducted research shows that epigenetic regulation is one of the important factors during these processes. The epigenetic environment within skeletal muscles modified with physical exercises precisely adjusts the delicate balance between gene expression and silencing under the control of contemporary constant transcriptional or post-transcriptional mechanisms [10,11].

### 1.2. DAT1

The human dopamine transporter gene (*DAT1* or *SLC6A3*) has been associated with various brain-related diseases and behavioral traits and, as such, has been investigated intensely in experimental- and clinical settings.

Dopamine (DA) neurotransmission underlies core brain functions, including locomotion, behavior, cognition, and motivation; consequently, disruption in dopamine signaling gives rise to various neuropsychiatric disorders and conditions [12]. A key player in the regulation of DA signaling is the dopamine transporter (DAT); it modulates the dynamics and the levels of DA in the synaptic cleft by recycling extracellular DA back into the presynaptic terminal. Alterations in the DAT availability in the brain directly affect the concentration of synaptic DA and the kinetics of its reuptake [13].

As our knowledge about methylation in particular promotor regions among athletes is still in its infancy, in hereto research, we presented its influence and correlation in connection with personality traits, simultaneously asking if the differences between individual personalities influence methylation of chosen promoter groups in the *DAT1* gene. We are aware that the causal relationship between personality traits and methylation processes can go both ways; hence we want to analyze the issue more precisely. Nonetheless, it needs more numerous groups.

## 2. Materials and Methods

### 2.1. Samples

The research group was 100 sports male subjects (mean age = 22.88, SD = 6.35), whereas the control group included 239 healthy male volunteers matched for age (mean age = 21.69, SD = 3.39). Both groups were composed of individuals of European origin from the same region of Poland. The research was based on 100 Polish healthy (no prior history of substance dependency or psychosis) male combat athletes (MMA, *n* = 23; judo, *n* = 40; boxing, *n* = 5; karate, *n* = 15; kickboxing, *n* = 15; wrestling, *n* = 2). Several methods were applied to prepare the samples, including targeting national teams and providing information to national coaching personnel and athletes attending training camps. All athletes and controls were European to reduce the possibility of genetic admixture and to overcome any potential problems due to population stratification.

The study was conducted according to the guidelines of the Declaration of Helsinki, and approved by KOMISJA BIOETYCZNA przy Okręgowej Izbie Lekarskiej w Szczecinie, ul. Marii Skłodowskiej-Curie 11, 71-332 (protocol nr 13/KB/VI/2016, 08.12.2016).

### 2.2. Methylation Status Assessment

Methylation of 33 promotor islands of the *DAT1* gene for the research and control group was accomplished and published previously by Michałowska-Sawczyn and coauthors [14].

DNA isolation kit (A&A Biotechnology, Gdynia, Poland) was used for DNA extraction from peripheral blood leukocytes. Extracted DNA was stored at −20 °C. Bisulfite modification of 250 ng DNA was accomplished with the usage of the EZ DNA Methylation Kit (Zymo Research, Orange, CA, USA), following the manufacturer’s instructions. Methylation-specific PCR assay was carried out in a Mastercycler ep gradient S (Eppendorf, Germany).

Primer oligonucleotides were obtained from Genomed.pl (Warsaw, Poland). Primer sequences were designed using MethPrimer (http://www.urogene.orgbin/methprimer/.cgi, accessed on 11 January 2020). The status of the DAT1 promoter (ENSG00000142319) was assessed by PCR using primers specific to a fragment of the gene, i.e., DATF: 5′-GGTTTTTGTTTTTTTTATTGTTGAG-3′; DATR: 5′-AAATCCCCTAAACCTAATCCC-3′. The PCR conditions in order to amplify the 447-bp fragment covering 33 CpG sites in DAT1 gene promoter were as follows: initial denaturation (94 °C/5 min), followed by 35 cycles (94 °C/61 °C/72 °C, 25 s each step) with final elongation at 72 °C for 5 min. The concentration of magnesium chloride ions was 2.5 mM. After amplification assay, the PCR products were subjected to sequencing as previously described [14]. Briefly, samples were verified by sequencing using the Bigdye v3.1 kit (Applied Biosystems, Darmstadt, Germany) and separation by ethanol extraction using the ABI Prism 3130XL (Applied Biosystems, Darmstadt, Germany) in a 36 cm capillary in a POP7 polymer, using the reverse primer in accordance with the manufacturer’s protocol.

Sequencing chromatograms were analyzed using 4peaks software (Mek and Tosj, Amsterdam, The Netherlands). Methylation of cytosine was considered positive, when the G/A + G ratio reached at least 20% of a total signal.

### 2.3. Assessment of the Ability to Bind Transcription Factors

For the analysis of transcription binding sites for the *DAT1* promoter region, we used PROMO software (http://alggen.lsi.upc.es/cgi-bin/promo_v3/promo/promoinit.cgi?dirDB = TF_8.3, accessed on 12 July 2021). In PROMO, for the identification of potential binding sites in sequences, weight matrices are constructed from known binding sites extracted from version 8.3 of the TRANSFAC database (http://genexplain.com/transfac/#section0, (11.01.2020), accessed on 12 July 2021). The ability of transcription factors to bind individual regions was assessed with different similarity rates, i.e., 100%, 95%, or 85%. As promotor sites showed to be *PAX5* transcription factor binding sites (positions 3, 22, and 33), and their hypomethylation showed to be important for personality traits, we searched for CpG status in relation with DAT1.

### 2.4. Psychometric Tests

Sports subjects and control subjects, both male, were examined by the NEO Five-Factor Personality Inventory (NEO-FFI) scales. The NEO Five-Factor Personality Inventory (NEO Five-Factor Inventory, NEO-FFI) includes 6 dimensions for each of the five traits–Extraversion (Positive Emotion, Warmth, Gregariousness, Activity, Excitement Seeking, Assertiveness), Agreeableness (Tender-mindedness, Trust, Altruism, Straightforwardness, Compliance, Modesty), Openness to experience (Fantasy, Feelings, Aesthetics, Actions, Values, Ideas), Conscientiousness (Deliberation, Competence, Dutifulness, Order, Achievement striving, Self-discipline), Neuroticism (Anxiety, Vulnerability to stress, Hostility, Self-consciousness, Impulsiveness, Depression) [15].

The results of NEO-FFI and STAI inventories were given as sten scores. The conversion of the raw score into the sten scale was performed according to Polish norms for adults; it was assumed that: sten 1–2—very low scores, 3–4—low scores, 5–6—average scores, 7–8—high scores, 9–10—very high scores.

### 2.5. Statistical Analysis

The relationship between DAT1 promotor methylation status, sports sand control subjects, and NEO Five-Factor Inventory (NEO-FFI) was analyzed by a multivariate analysis of Factor effects ANOVA (NEO-FFI× methylation status × sports sand control subjects × (methylation status × sports sand control subjects)). Not all assumptions required for the ANOVA analysis have been met. The assumption about the normal distribution was not fulfilled for all dependent variables, but the variance was the same (Levene’s test *p* > 0.05). Because the number of subjects in groups was also large, it was therefore decided to use multivariate analysis 2 × 3 factorial ANOVA. The NEO Five-Factor Inventory (Neuroticism, Extraversion, Openness, Agreeability Conscientiousness) was measured and compared using the U Mann-Whitney test. Methylation status data was analyzed using a chi-square test with a *p* < 0.05 being considered statistically significant. The whole process of calculation was performed with the usage of STATISTICA 13 (Tibco Software Inc, Palo Alto, CA, USA) for Windows (Microsoft Corporation, Redmond, WA, USA).

## 3. Results

The methylation status of the CpG PAX5 4 *DAT1* promotor sites (3, 13, 22, 33) in sports and control subjects is shown in Table 1.

The means and standard deviations for all NEO Five-Factor Inventory and interaction for sports subjects and control subjects are presented in Table 2.

When comparing the controls and the study group subjects, for the second one, we observed significantly higher scores (Table 2) on the NEO Five-Factor Inventory scale of Conscientiousness (M 7.23 vs. M 5.83, *p* < 0.0001).

### 3.1. PAX 5 CpG Position: Sites 3

**Figure 1:**
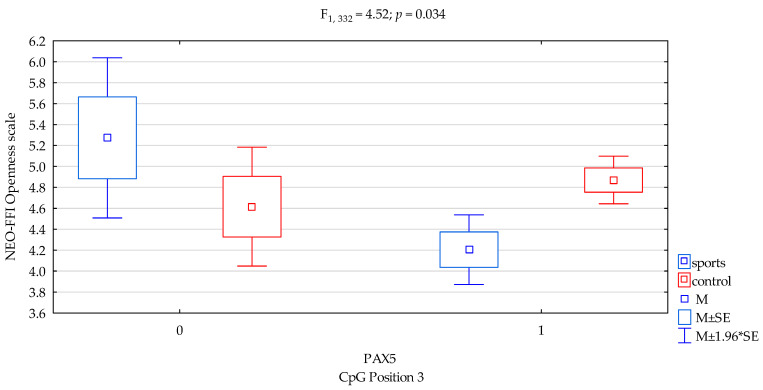
Interaction between sports subjects/control and *DAT1* PAX5 CpG sites 3 and NEO FFI Openness scale. Methylation status: yes—1, no—0. M—mean, M ± SE—mean ± standard error, M ± 1.96*SE—mean ± s1.96*standard error.

The results of 2 × 3 factorial ANOVA of the NEO Five-Factor Personality Inventory (NEO-FFI) sten scales and *DAT1* promotor methylation status of the CpG PAX5 sites 3 and controls and the study group subjects are summarized in Table 3. When comparing groups, we found a significant result in the interactions (sports subjects vs. controls) for the NEO FFI Openness scale and *DAT1* promotor methylation status of the CpG PAX5 sites 3 (Figure 1, F_2,332_ = 4.52, *p* = 0.034), accounting for 1.3% of the variance, respectively. The results of the post hoc test are included in Table 4.

### 3.2. PAX 5 CpG Position: Sites 13

**Figure 2:**
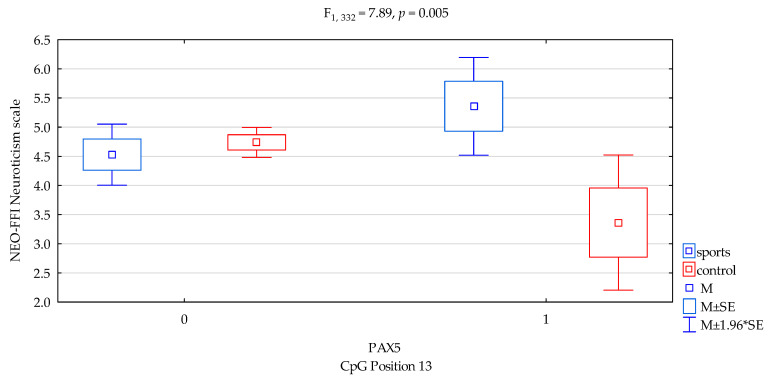
Interaction between sports subjects/control and *DAT1* PAX5 CpG sites 13 and NEO FFI Neuroticism scale. Methylation status: yes—1, no—0. M ± SE—mean ±standard error, M ± 1.96*SE—mean ± s1.96*standard error.

The results of 2 × 3 factorial ANOVA of the NEO Five-Factor Personality Inventory (NEO-FFI) sten scales and *DAT1* promotor methylation status of the CpG PAX5 sites 13, controls, and the study group subjects are summarized in Table 3. We found interactions a significant result when comparing groups (sports subjects vs. controls) for NEO FFI Neuroticism and *DAT1* promotor methylation status of the CpG PAX5 sites 13 (Figure 2, F_2,332_ = 7.89, *p* = 0.005), accounting for 2.3% of the variance, respectively. The results of the post hoc test are included in Table 4.

### 3.3. PAX 5 CpG Position: Sites 22

The results of 2 × 3 factorial ANOVA of the NEO Five-Factor Personality Inventory (NEO-FFI) sten scales and *DAT1* promotor methylation status of the CpG PAX5 sites 13, controls, and the study group subjects are summarized in Table 3.

### 3.4. Neuroticism Scale

**Figure 3:**
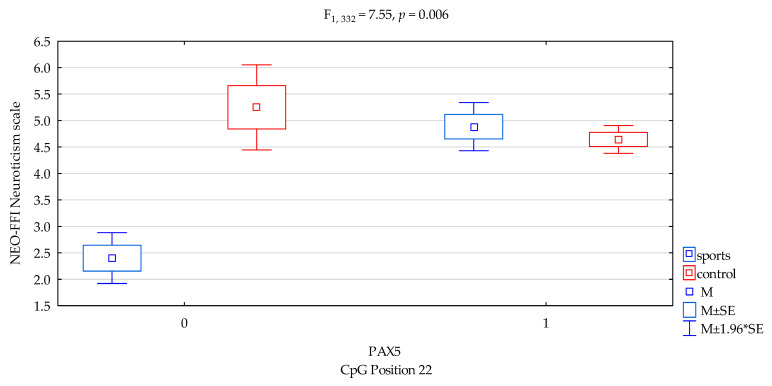
Interaction between sports subjects/control and *DAT1* PAX5 CpG sites 22 and NEO FFI Neuroticism scale. Methylation status: yes—1, no—0. M ± SE—mean ±standard error, M ± 1.96*SE—mean ± s1.96*standard error.

We found interactions a significant result when comparing groups (sports subjects vs. controls) for NEO FFI Neuroticism, and *DAT1* promotor methylation status of the CpG PAX5 sites 22 (Figure 3, F_2,332_ = 7.55, *p* = 0.006), accounting for 2.2% of the variance, respectively. The results of the post hoc test are included in Table 4.

### 3.5. Extraversion Scale

**Figure 4:**
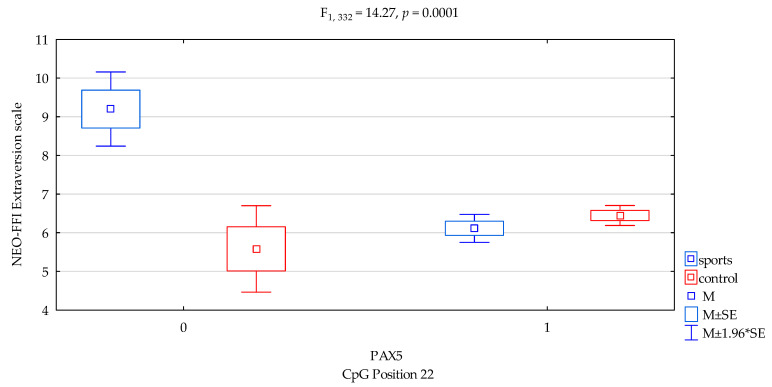
Interaction between sports subjects/control and *DAT1* PAX5 CpG sites 22 and NEO FFI Extraversion scale. Methylation status: yes—1, no—0. M ± SE—mean ±standard error, M ± 1.96*SE—mean ± s1.96*standard error.

We found a significant result when comparing for NEO-FFI Extraversion scale for *DAT1* promotor methylation status of the CpG PAX5 sites 22 (F_2,332_ = 4.52, *p* = 0.0342), accounting for 1.3% of the variance, respectively. We found interactions a significant result when comparing groups (sports subjects vs. controls) for NEO FFI Extraversion and DAT1 promotor methylation status of the CpG PAX5 sites 22 (Figure 4, F_2,332_ = 14.27, *p* = 0.0001), accounting for 4.1% of the variance, respectively. The results of the post hoc test are included in Table 4.

### 3.6. Openness Scale

**Figure 5:**
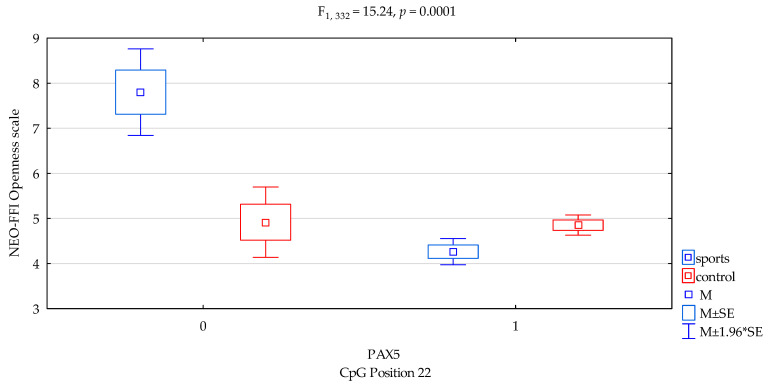
Interaction between sports subjects/control and *DAT1* PAX5 CpG sites 22 and NEO FFI Openness scale. Methylation status: yes—1, no—0. M ± SE—mean ±standard error, M ± 1.96*SE—mean ± s1.96*standard error.

We found a significant result when comparing for NEO-FFI Openness scale for *DAT1* promotor methylation status of the CpG PAX5 sites 22 (F_2,332_ = 16.39, *p* = 0.0001), accounting for 4.7% of the variance, respectively. We found interactions a significant result when comparing groups (sports subjects vs. controls) for NEO FFI Openness and *DAT1* promotor methylation status of the CpG PAX5 sites 22 (Figure 5, F_2,332_ = 15.24, *p* = 0.0001), accounting for 4.4% of the variance, respectively. The results of the post hoc test are included in Table 4.

### 3.7. Agreeability Scale

**Figure 6:**
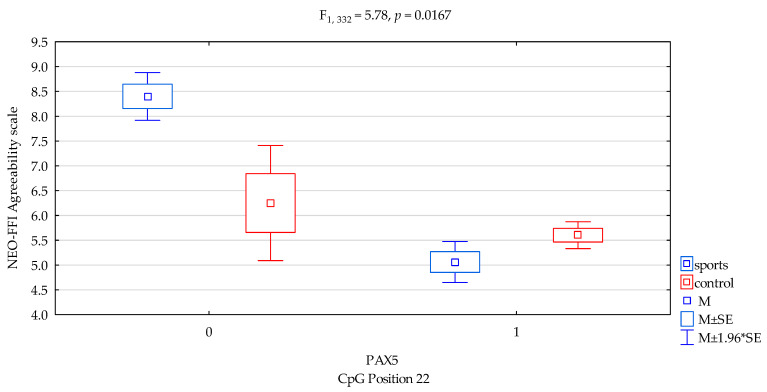
Interaction between sports subjects/control and *DAT1* PAX5 CpG sites 22 and NEO FFI Agreeability scale. Methylation status: yes—1, no—0. M ± SE—mean ±standard error, M ± 1.96*SE—mean ± s1.96*standard error.

We found a significant result when comparing for NEO-FFI Agreeability scale for *DAT1* promotor methylation status of the CpG PAX5 sites 22 (F_2,332_ = 12.68, *p* = 0.0004), accounting for 3.7% of the variance, respectively. We found interactions a significant result when comparing groups (sports subjects vs. controls) for NEO FFI Agreeability and *DAT1* promotor methylation status of the CpG PAX5 sites 22 (Figure 6, F_2,332_ = 5.78, *p* = 0.0167), accounting for 1.7% of the variance, respectively. The results of the post hoc test are included in Table 4.

### 3.8. PAX 5 CpG Position: Sites 33

We did not find significant results for the 2 × 3 factorial ANOVA of the NEO Five-Factor Personality Inventory (NEO-FFI) sten scales, the *DAT1* methylation status of the CpG PAX5 sites 33 and controls and the study group subjects.

## 4. Discussion

### DAT1 and Methylation

Experimental and clinical evidence indicates that sequence variations upstream of the transcription start site (TSS) affect *DAT1* transcriptional regulation [16]. The *DAT1* core promoter lacks “TATA” and “CAT” boxes (these DNA sequences provide docking sites for basal transcriptional complex) [17]. Transcriptional initiation of neuronal genes often is facilitated via binding of core transcriptional machinery to the CCAAT element [18]. Shumay et al. [19] detected several CCAAT consensuses upstream and downstream of the *DAT1* TSS, suggesting that the *DAT1* might be one of these. Human genes that have CCAAT-promoters display several common characteristics; in general, they are less precise in terms of TSS than the genes with TATA-promoters, and they mostly overlap with CpG islands. Transcription initiation from the CCAAT box involves NF-Y, an element with histone-like features, and a particular subset of transcription factors [20].

The complex structure of human gene promoters with a range of alternative transcription start sites (TSSs) [21] supports differential temporal- and spatial- patterns of gene expression and provides an additional level of gene regulation by modulating translational efficiency [22]. About one-fifth of human genes have alternative promoters; this phenomenon is most frequent in brain-related genes [23].

The interaction of the cis-regulatory elements of a gene with transcription factors (TF) largely determined transcription events; therefore, an assessment of the putative TF binding sites in the regulatory region of the gene under analysis yields important information on this gene’s regulation. In the nervous system, TFs define the basic framework; their availability varies across the brain regions and cell types, thus contributing to phenotypic diversity [24]. As we mentioned, the nature of the *DAT1* promoter (CCAAT promoter) implies its sensitivity to selective TFs, because NF-Y synergistically interacts with a subset of TFs [20]. Shumay et al. [19] inspection of the DAT1 5′-flanking sequence (−2 kb, JASPAR database) revealed that it contains binding sites for Sp1, GATA−1, CREB, and c-Myc cis-acting regulatory elements –all those TFs interact with NF-Y [20].

The human *DAT1* gene is remarkably GC dense and has multiple CpG islands: in contrast to most vertebrate genes that have only a promoter-overlapping CpG Island. On average, inactive promoters of the human genes cytosine and guanine account for 57% of the nucleotides [25], but GCs represent 79% of the *DAT1* promoter sequence.

In vivo, the DNA molecule forms a complex with proteins that allow its packaging into chromatin. Nucleosomes are the structural units of chromatin represented by histone octamers around which the DNA coils. The close interaction of the DNA molecule with a nucleosome core results in condensed chromatin that is inaccessible to the transcription machinery; hence, the transcriptional activation of a gene requires the local transition of compact chromatin domains into decondensed loops. Nucleosome remodeling and covalent modifications of histones provide the basis for epigenetic gene regulation that occurs via the modulation of the accessibility of the genomic loci to transcriptional machinery [26]. CG-rich motifs in DNA sequences inherently disfavor nucleosomes and are referred to as “nucleosome exclusion sequences” (NX) [27]. Shumay et al. [19] found that both the *DAT1* and the 5-HTT (*SLC6A4*) genes have high NX-scoring sequences near the TSS. The predicted nucleosome positioning in the *DAT1* and the *5-HTT* loci notably differ: the entire DAT1 locus comprises of numerous nucleosome-dysfavouring sequences, while in the *5-HTT*, NX-peaks are sparse. It was suggested that intragenic regions with high NX Scores might function as transcriptional enhancers.

The most notable characteristic of the human *DAT1* is its high sensitivity to epigenetic regulation: in contrast to the relative enrichment in GC nucleotides in the promoter-proximal region as occurs in most human genes, the entire *DAT1* locus has GC-bias sequence composition (0.55) and comprises multiple CpG sites comprising 27 bona fide CGIs (CpG islands). [19].

Previous studies found that *DAT1* methylation derived from blood correlated with symptoms of hyperactivity and impulsivity in children and adolescents with ADHD [28] and with impulsivity (and basal ganglia DAT availability) in monkeys [29]. Hence, in our research, we combined methylation with personality traits. In the presented research, we noticed that in *DAT1* PAX5, CpG island 3 observed a statistically significant interaction between the occurrence of methylation in martial arts athletes and lower results in sten scale of NEO FFI Openness in comparison with the control group (Figure 1, 4.20 vs. 4.87, *p* = 0.0026, Table 4). In *DAT1* PAX5, CpG island 13 observed a statistically significant interaction between methylation in martial arts athletes and increased results in sten scale NEO FFI Neuroticism compared to the control group (Figure 2, 5.36 vs. 3.36, *p* = 0.0069, Table 4). The part of the research shows a distinct occurrence of methylation in these subgroups of athletes. However, we observed additionally that in particular subgroups based on the personality traits tests, methylation does not occur. Still, in *DAT1* PAX5, CpG island 22 noticed statistically significant interaction between the shortage of methylation in the martial arts athletes and lower results in sten scale NEO FFI Neuroticism in comparison with the control group (Figure 3, 2.40 vs. 5.25, *p* = 0.0098, Table 4). In *DAT1* PAX5, CpG island 22 noticed statistically significant interaction between the shortage of methylation in the martial arts athletes and increased results in sten scale NEO FFI Openness compared to the control group (Figure 5, 7.80 vs. 4.92, *p* = 0.0004, Table 4). However, reverse interaction was observed in the case of methylation; subjects from the martial arts athletes group obtained lower results in sten scale NEO FFI Openness compared to the control group (Figure 5, 4.26 vs. 4.85, *p* = 0.0033, Table 4).

In *DAT1* PAX5, CpG island 22 noticed statistically significant interaction between the shortage of methylation in the martial arts athletes and increased results in sten scale NEO FFI Agreeability compared to the control group (Figure 6, 8.40 vs. 6.25, *p* = 0.0494, Table 4). However, reverse interaction was observed in the case of methylation; subjects from the martial arts athletes group obtained lower results in sten scale NEO FFI Agreeability compared to the control group (Figure 6, 5.06 vs. 5.60, *p* = 0.0322, Table 4).

Epigenetic sensitivity of the *DAT1* gene increased during the process of evolution. The genetic drift of the *DAT1* sequence oriented on the accumulation of GC nucleotides may reflect its strengthening epigenetic potential, important in the regulatory processes resulting from more and more complex functions of the human brain [19].

We also want to emphasize that the discussed PAX5 (BSAP) functions as both a transcriptional activator and repressor during midbrain patterning, B-cell development, and lymphomagenesis [30].

In spite of the same genetic make-up of all cells of an organism, each tissue, and even each cell, has its own methylation pattern that determines its identity and functions as a result of dynamic interactions with other cells and the environment. Environmental stimuli through the mediation of several elements/factors such as neurotransmitters, hormones, and transcription factors modulate promoter methylation patterns and corresponding expression levels of various genes. Since successive bindings of transcription factors to a gene’s regulatory regions are associated with a decrease in DNA methylation level and an increase in the capability of gene expression for a prolonged period of time in the future, DNA methylation is considered as a mechanism for cell memory. The pattern of DNA methylation is generally maintained throughout cell division; therefore, any DNA methylation changes could either be global in the entire genome affecting all progenies of the affected cell or local, affecting only specific genes in specific cells [31].

Although the effects of gene methylation on gene expression are complex, gene methylation is generally seen as a ‘silencing’ epigenetic mark. That is, various studies have found that methylation of CpG islands in the promoter area has an inhibitory effect on transcription initiation, resulting in reduced gene expression [32,33]. The observed association between the methylation of *DAT1* and its expression might reflect the high concentration of CpG islands in the gene, which makes DAT expression particularly susceptible to modulation through epigenetic mechanisms, specifically DNA methylation [19]. Because DNA methylation is a dynamic measure, it might, however, better reflect the expression of proteins sensitive to modification by environmental exposures. The expression of *DAT1* is dynamic and sensitive to circadian rhythms [34], age [35], addictive drugs including tobacco [36,37,38], medication exposures [39], among others. Although multiple factors regulate DAT expression, methylation of *DAT1* is of particular interest as it changes dynamically in response to various environmental influences [19].

## 5. Conclusions

Although the effects of gene methylation on gene expression are complex, gene methylation is generally seen as a ‘silencing’ epigenetic mark. When analyzing genetic conditioning or associations in sport, it is also important to take into consideration the factors connected with the functioning of the human brain. Individual personality traits can differ in the area of methylation factors’ sensitivity. However, the tested group and the number of tested promotor islands in the *DAT1* gene are still too small to make explicit conclusions, so it still needs further analysis.

## Figures and Tables

**Table 1:** Methylation status of PAX5 4 DAT1 CpG sites (3, 13,22,33) in sports subjects and control group. A group of 100 sports and 239 control individuals were studied to compare methylation status in indicated CpG sites. Chi-square test χ^2^(p), Chi-square; OR, odds ratio; CI, Confidence Interval; (−95%, +95%) [14].

CpG Site	Studied Group	Methylation Status (%)	χ^2^(p)	OR	95% CI (−95%, +95%)
3 *	sports subjects N (100)	78%	20.471 (0.00001)	4.838	(2.326; 10.065)
	control N (239)	94%			
13 *	sports subjects N (100)	28%	37.290 (0.00001)	0.126	(0.059; 0.265)
	control N (239)	5%			
22	sports subjects N (100)	95%	0.001 (0.974)	0.982	(0.337; 2.865)
	control N (239)	95%			
33	sports subjects N (100)	66%	9.291 (0.0023)	2.247	(1.326; 3.810)
	control N (239)	81%			

* Significant between-group differences.

**Table 2:** NEO Five-Factor Inventory results (sten scale) and between healthy control and sports subjects.

STAI/NEO Five Factor Inventory/	Sports Subjects (N = 100)	Control (N = 236)	Z	*p* Value
Neuroticism/scale	4.76 ± 2.28	4.66 ± 1.99	0.145	0.884
Extraversion/scale	6.27 ± 1.89	6.41 ± 1.97	−0.692	0.488
Openness/scale	4.44 ± 1.63	4.83 ± 1.70	−1.531	0.126
Agreeability/scale	5.23 ± 2.13	5.65 ± 2.07	−1.428	0.153
Conscientiousness/scale	7.23 ± 1.86	5.83 ± 2.13	5.498	0.0000 *

*p*-statistical significance U Mana’s test, N—number of subjects, M ± SD—Mean ± Standard Deviation. *—Significant between-group differences.

**Table 3:** Differences in methylation *DAT1* PAX5 CpG sites 3, 13, 22, 33 and NEO Five-Factor Inventory between healthy control subjects and sports subjects.

STAI/NEO Five-Factor Inventory/					ANOVA				
	Sports (N = 100) M ± SD	Control (N = 236) M ± SD	Methylation Status No (N = 17) M ± SD	Methylation Status Yes (N = 319) M ± SD	Full Model F *p* Value R^2^	Factor	F (*p* Value)	ɳ^2^	Power (alfa = 0.05)
PAX 5 CpG Position: sites 3									
Openness/scale	4.44 ± 1.63	4.83 ± 1.70	5.02 ± 1.60	4.70 ± 1.69	F_3,332_ = 3.913 *p* = 0.0091 * R^2^ = 0.034	intercept	F_1,332_ = 929.65 (*p* < 0.0001)	0.737	1.000
						sports/control	F_1,332_ = 0.0001 (*p* = 0.990)	0.00001	0.050
						CpG sites 3	F_2,332_ = 1.71 (*p* = 0.192)	0.005	0.256
						addicts/control x CpG sites 3	F_2,332_ = 4.52 (*p* = 0.034) *	0.013	0.563
PAX 5 CpG Position: sites 13									
Neuroticism/scale	4.76 ± 2.28	4.66 ± 1.99	4.69 ± 2.04	4.79 ± 2.34	F_3,332_ = 2.687 *p* = 0.0465 * R^2^ = 0.024	intercept	F_1,332_ = 526.07 (*p* < 0.0001) *	0.613	1.000
						sports/control	F_1,332_ = 5.17 (*p* = 0.024) *	0.015	0.621
						CpG sites 13	F_2,332_ = 0.48 (*p* = 0.488)	0.001	0.107
						addicts/control x CpG sites 13	F_2,332_ = 7.89 (*p* = 0.005) *	0.023	0.800
PAX 5 CpG Position: sites 22									
Neuroticism/scale	4.76 ± 2.28	4.66 ± 1.99	4.41 ± 1.80	4.71 ± 2.08	F_5,332_ = 2.674 *p* = 0.0473 * R^2^ = 0.024	intercept	F_1,332_ = 233.11 (*p* < 0.0001) *	0.412	1.000
						sports/control	F_1,332_ = 5.38 (*p* = 0.0210) *	0.016	0.638
						CpG sites 22	F_2,332_ = 2.78 (*p* = 0.0962)	0.008	0.383
						addicts/control x CpG sites 22	F_2,332_ = 7.55 (*p* = 0.006) *	0.022	0.782
Extraversion/scale	6.27 ± 1.89	6.41 ± 1.97	6.65 ± 2.42	6.35 ± 1.92	F_5,332_ = 5.001 *p* = 0.0021 * R^2^ = 0.0432	intercept	F_1,332_ = 685.04 (*p* < 0.0001) *	0.673	1.000
						sports/control	F_1,389_ = 9.89 (*p* = 0.0018) *	0.029	0.880
						CpG sites 22	F_2,389_ = 4.52 (*p* = 0.0342) *	0.013	0.563
						addicts/control x CpG sites 22	F_2,389_ = 14.27 (*p* = 0.0001) *	0.041	0.965
Openness/scale	4.44 ± 1.63	4.83 ± 1.70	5.76 ± 1.86	4.68 ± 1.66	F_5,332_ = 9.003 *p* = 0.00001 * R^2^ = 0.075	intercept	F_1,332_ = 602.61 (*p* < 0.0001) *	0.644	1.000
						sports/control	F_1,332_ = 6.65 (*p* = 0.0103) *	0.020	0.729
						CpG sites 22	F_2,332_ = 16.39 (*p* = 0.0001) *	0.047	0.981
						addicts/control x CpG sites 22	F_2,332_ = 15.24 (*p* = 0.0001) *	0.044	0.973
Agreeability/scale	5.23 ± 2.13	5.65 ± 2.07	6.88 ± 1.99	5.44 ± 2.07	F_5,332_ = 5.500 *p* = 0.0011 * R^2^ = 0.047	intercept	F_1,332_ = 512.12 (*p* < 0.0001) *	0.607	1.000
						sports/control	F_1,332_ = 2.07 (*p* = 0.1509) *	0.006	0.300
						CpG sites 22	F_2,332_ = 12.68 (*p* = 0.0004) *	0.037	0.944
						addicts/control x CpG sites 22	F_2,332_ = 5.78 (*p* = 0.0167) *	0.017	0.669

M—mean, SD—standard deviation. *—Statistically significant between-group differences.

**Table 4:** Post hoc analysis of interactions between sports subjects/control and *DAT1* PAX5 CpG sites 3, 13, 22, 33 and NEO FFI scale.

*DAT1* PAX5 CpG Sites 3 Openness Scale				
	{1} M = 5.27	{2} M = 4.20	{3} M = 4.62	{4} M = 4.87
Sport; methylation status no {1}		0.0082 *	0.2596	0.2796
Sport; methylation status yes {2}			0.4111	0.0026*
Control; methylation status no {3}				0.5922
Control; methylation status yes {4}				
*DAT1* PAX5 CpG sites 13 Neuroticism scale				
	{1} M = 4.53	{2} M = 5.36	{3} M = 4.74	{4} M = 3.36
Sport; methylation status no {1}		0.07151	0.4520	0.0817
Sport; methylation status yes {2}			0.1344	0.0069 *
Control; methylation status no {3}				0.0314 *
Control; methylation status yes {4}				
*DAT1* PAX5 CpG sites 22 Neuroticism scale				
	{1} M = 2.40	{2} M = 4.88	{3} M = 5.25	{4} M = 4.64
Sport; methylation status no {1}		0.0090 *	0.0098 *	0.0166 *
Sport; methylation status yes {2}			0.5625	0.3393
Control; methylation status no {3}				0.3206
Control; methylation status yes {4}				
*DAT1* PAX5 CpG sites 22 Extraversion scale				
	{1} M = 9.20	{2} M = 6.12	{3} M = 5.58	{4} M = 6.45
Sport; methylation status no {1}		0.0005 *	0.0004 *	0.0016 *
Sport; methylation status yes {2}			0.3642	0.1590
Control; methylation status no {3}				0.1288
Control; methylation status yes {4}				
*DAT1* PAX5 CpG sites 22 Openness scale				
	{1} M = 7.80	{2} M = 4.26	{3} M = 4.92	{4} M = 4.85
Sport; methylation status no {1}		0.0000 *	0.0010 *	0.0001 *
Sport; methylation status yes {2}			0.1911	0.0033 *
Control; methylation status no {3}				0.8946
Control; methylation status yes {4}				
*DAT1* PAX5CpG sites 22 Agreeability scale				
	{1} M = 8.40	{2} M = 5.06	{3} M = 6.25	{4} M = 5.60
Sport; methylation status no {1}		0.0004	0.0494 *	0.0027 *
Sport; methylation status yes {2}			0.0594	0.0322 *
Control; methylation status no {3}				0.2869
Control; methylation status yes {4}				

*—significant statistical differences, M—mean.

## Data Availability

Not applicable.
